# Management of Infants Treated for Respiratory Viral Infections: A Finnish Retrospective Register‐Based Study

**DOI:** 10.1002/hsr2.71414

**Published:** 2025-10-28

**Authors:** Sallamaria Länsisalo, Paula Heikkilä, Sauli Palmu

**Affiliations:** ^1^ Faculty of Medicine and Health Technology Tampere University Tampere Finland; ^2^ Tampere Centre for Child, Adolescent and Maternal Health Research, Faculty of Medicine and Health Technology Tampere University and University Hospital Tampere Finland; ^3^ Department of Pulmonology, Dermatology and Allergology Tampere University Hospital, Wellbeing Services County of Pirkanmaa Tampere Finland; ^4^ Department of Pediatrics Tampere University Hospital, Wellbeing Services County of Pirkanmaa Tampere Finland

**Keywords:** fever, respiratory infection, viral infection

## Abstract

**Background and Aims:**

Viral respiratory tract infections are common in all age groups, and they are responsible for a vast number of health care visits every year. Furthermore, the incidence of respiratory infections is the highest in children. We aimed to determine how infants presenting with fever and symptoms of respiratory infections were treated at Tampere University Hospital (Tays) and their responses to treatment. The secondary purpose was to describe the specific diagnosis behind the respiratory symptoms.

**Methods:**

This study was conducted in the Department of Paediatrics in Tays, Finland. Inclusion criteria were age < 1 year, fever, and symptoms of a respiratory infection. We documented from the electronic patient records the medication and other treatments the infants received during their hospital stays. For infants given the same medication multiple times, only the first dosing and response to that medication were documented.

**Results:**

The study included 119 episodes (117 infants), 50 (42%) of whom were hospitalized. Symptom‐relieving medications, such as paracetamol, were used frequently, with generally good responses. Overall, 59% of the infants were administered antibiotics, the most common indication being otitis media. The most common primary or secondary diagnosis was bronchiolitis or bronchitis (43%).

**Conclusion:**

Nearly half of the infants needed hospitalization. Symptom‐relieving medications, such as analgesics, were a key treatment choice, and the responses were generally good. Although the majority of the infants had a viral infection, antibiotics were used frequently, at least partially due to secondary bacterial infections.

**Abbreviations**
CRP: C‐reactive protein
ED: emergency department
RSV: respiratory syncytial virus
Tays: Tampere University Hospital

## 1 Introduction

Viral respiratory tract infections are common in all age groups, and they are responsible for a vast number of health care visits every year. Furthermore, the incidence of respiratory infections is the highest in children [1]. Symptoms of upper respiratory tract infections in children include cough, running nose, soreness, and hoarseness of throat, and fatigue [2]. In addition to these symptoms, respiratory infections can cause fever in children [2, 3]. All respiratory viruses can also cause infections in the lower respiratory tract, and some viruses tend to cause specific infections [3]. For instance, respiratory syncytial virus (RSV) is the most common cause of bronchiolitis in infants [4]. It is not uncommon that viral upper respiratory tract infections are also complicated by secondary infections, such as acute otitis media (AOM) or pneumonia, which are often at least of partially bacterial etiology [2]. For example, it is estimated that up to 75% of children with influenza infection develop AOM [5]. This study is focused to the treatment of infants presenting at the emergency department (ED) with symptoms of upper or lower respiratory tract infection.

Treatment guidelines for different respiratory tract infections and their symptoms vary [3]. Currently, specific antiviral medications are available only for influenza virus infections. The treatment of other viral respiratory infections is mainly focused on relieving symptoms [2]. Antibiotic treatment is advocated only if there is a concomitant bacterial infection [4]. The purpose of this study was to determine how children aged < 1 year presenting with fever and symptoms of a respiratory infection were treated, as well as the response to the treatment at Tampere University Hospital (Tays) Department of Paediatrics. The secondary purpose was to describe the specific diagnosis causing the respiratory symptoms.

## 2 Materials and Methods

This descriptive, retrospective study was conducted in the pediatric unit of Tampere University Hospital, Finland. At the previous stage of the study, the infants included were identified from a prospective population‐based epidemiological study conducted in the ED of Tampere University Hospital (Tays), Finland [6]. During the study period, 4386 children under the age of 1 year lived in the catchment area of Tays.

As described previously, our study included infants who visited the ED during December 27, 2019 to May 6, 2020. Inclusion criteria were age < 1 year, fever (≥ 38°C), and at least one symptom of a respiratory infection, such as cough, fatigue, running nose, coryza, or general discomfort [6]. From infants who met these inclusion criteria, a nasal swab specimen was collected and tested for influenza A and B viruses as well as for RSV. In the present study, one of the authors (S. L.) collected retrospectively background and medical data relating to outpatient and inpatient episodes from Tays' electronic patient records. Demographic information gathered included infants' age on admission, gender, gestational age at birth, weight and height at birth, allergies and chronic illnesses, home medication, and history of hospital treatment after birth. Information about infants' respiratory rates, lung auscultation sounds, and possible dehydration was documented as the infants arrived at the ED. The highest values of body temperature and laboratory results (C‐reactive protein [CRP] and leukocytes) during the entire time of the infant's inpatient episode were documented. Our study hospital's reference value for CRP was < 5 mg/L, and CRP values that were announced as < 5 mg/L were reported as 5 mg/L. All chest radiographs obtained during the time of the infant's hospitalization were documented.

We also recorded the medications and other treatments that were given to infants during their hospital stays as well as infants' response to the medication. The response was evaluated by hospital staff as part of their daily routines. We documented whether the response was registered for the patient records, and if it was considered good or nonexistent. Considering analgesics, response was considered good if hospital staff documented a favorable effect, for example, that infant was calmed down or slept peacefully after receiving pain medication. If an infant was given analgesics, antipyretics, or inhaled medications multiple times, only the first dose and response were documented. For antibiotics and oseltamivir, the treatment response was considered favorable if there was no need to switch to a different medication, and for patients treated as outpatients, the response was considered favorable if they did not need to return to the ED during the same infection episode. A treatment response was considered favorable if fluid therapy, oxygen support, and high‐flow nasal cannula respiratory support were sufficient without the need to escalate to another treatment modality, and if these treatments could eventually be discontinued. In addition, the length of stay in the hospital was calculated for inpatients.

Information was gathered from the hospital's patient records and families were not contacted for this study. Therefore, informed consent from patients or their parents was not required according to Finnish law.

### 2.1 Statistical Methods

The results are presented by number and percentages in categorical variables and by median and lower and upper quartiles (Q_1_, Q_3_) in continuous variables, as the data were non‐normally distributed. To explore potential differences between the virus groups, we performed subgroup analyses. In the analyses, one virus group was always compared to all other cases. Infants that did not test positive for influenza A or B viruses or RSV were categorized to “other etiology” group for statistical analysis. In this group, the viral cause for infection was left unknown. The Mann–Whitney *U* test was used to compare continuous data of age, gestational age at birth, weight, and height at birth, length of hospitalization, and laboratory values. Proportions of categorical variables such as gender, hospitalization, prematurity, lung auscultation sounds, and treatments were compared using the *χ* ^2^ test or Fisher's exact test. All the tests were two‐sided. Since we performed multiple subgroup comparisons, we applied the Bonferroni adjustment, and accordingly, a *p* value < 0.017 was considered statistically significant. Statistical analyses were performed with IBM Corp. SPSS Statistics, version 26.

## 3 Results

In total, 119 episodes (117 infants) met the inclusion criteria, and two infants had two separate infection episodes (Table 1). Of all cases, 66 (56%) were boys. Most of the infants were born full‐term, with a median gestational age of 39 weeks. The median weight at birth was 3540 g. There were 21 infants (18%) who needed hospital treatment as newborns. None of the infants had any permanent medications (Table 1). During the study period, 50/119 patients (42%) were hospitalized, and of those, one case (2%) was treated in the intensive care unit (ICU). The median length of stay in hospital was 2 days (Q_1_–Q_3_ 1.8–4) (Table 1). The most common primary or secondary diagnoses were bronchiolitis or bronchitis (43%), otitis media (38%), and upper respiratory tract infections (27%). Within hospitalized infants, the proportion that were diagnosed with bronchiolitis or bronchitis was even higher, at 56% (Supporting Material S1).

**Table 1:** Background data and characteristics of the hospital stay of the 117 infants (119 treatment episodes) treated for fever and symptoms of respiratory infection at the Pediatric Unit of Tampere University Hospital between December 27, 2019 and May 6, 2020.

	All cases	Influenza	RSV	Other etiology
Number of cases, *n* (%)	119	11 (9.2)	66 (55)	44 (37)
Age, months, median (Q_1_–Q_3_)	6.1 (2.9–9.1)	2.9 (1.3–8.3), *p* = 0.09	6.6 (3.1–9.4), *p* = 0.5	6.6 (2.8–9.2), *p* = 0.8
Boys, *n* (%)	66 (55)	4 (36), *p* = 0.2	38 (58), *p* = 0.6	24 (55), *p* = 0.9
Gestational age at birth, weeks, median (Q_1_–Q_3_)^a^	39 (38–40)	39 (38–40), *p* = 0.7	39 (38–40), *p* = 0.9	39 (37–40), *p* = 0.9
Weight at birth, g, median (Q_1_–Q_3_)^a^	3540 (3166–3983)	3550 (3120–4010), *p* > 0.99	3590 (3195–4053), *p* = 0.2	3435 (2999–3816), *p* = 0.1
Palivizumab prophylaxis for RSV	0	0	0	0
Needed hospital treatment after birth, *n* (%)^a^	21 (18)	2 (18)	12 (18)	8 (18)
Permanent medication, *n* (%)	0	0	0	0
Chronic illness, *n* (%)	3 (2.5)	1 (9.1), *p* = 0.3	0 (0), *p* = 0.09	2 (4.5), *p* = 0.6
Allergy, *n* (%)	3 (2.5)	0 (0), *p* > 0.99	2 (3.0), *p* > 0.99	1 (2.3), *p* > 0.99
Hospitalization, *n* (%)	50 (42)	4 (36), *p* = 0.8	30 (45), *p* = 0.4	18 (41), *p* = 0.9
ICU^b^ treatment, *n* (%)	1 (2.0)	0, *p* > 0.99	0, *p* = 0.4	1 (5.6), *p* = 0.4
Length of stay, days, median (Q_1_–Q_3_)	2 (1.8–4)	2 (2–6), *p* = 0.8	2.5 (2–4), *p* = 0.2	2 (1–3.5), *p* = 0.2
Repeat appointments, *n* (%)	10 (8.4)	1 (9.1), *p* > 0.99	6 (9.1), *p* > 0.99	3 (6.8), *p* = 0.7
Number of repeat appointments, median (Q_1_–Q_3_)	1.0 (1.0–1.0)	1, *p* > 0.99	1 (1.0–1.25), *p* = 0.8	1 (1.0–1.0), *p* = 0.8

^a^ Information was missing from 1 case.

^b^ ICU = intensive care unit.

Upon arrival at the hospital, 52/119 infants (44%) had abnormal lung auscultation sounds (Table 2). The most common abnormal finding was coarse crackles (*n* = 42, 81%). Among the different etiology groups, abnormal lung auscultation sounds were most often found in infants who had an RSV infection. No infants had apnea at their arrival at the hospital, but 25/119 (21%) had signs of respiratory distress, such as retractions in the intercostal muscles (Table 2). Chest radiographs of 21/119 infants (18%) were taken, and out of those, 12/21 (57%) had abnormal findings, which most commonly were pneumonic infiltrations. In total, 7/119 (5.9%) infants had clinical signs of dehydration (Table 2).

**Table 2:** Clinical findings of the infants upon arrival at the emergency department.

	All cases (*n* = 119)	Influenza (*n* = 11)	RSV (*n* = 66)	Other etiology (*n* = 44)
Abnormal lung auscultation, *n* (%)	52 (44)	3 (27), *p* = 0.3	36 (55), *p* = 0.08	15 (34), *p* = 0.1
Wheezing, *n* (%)	20 (38)	0 (0), *p* = 0.3	16 (44), *p* = 0.2	4 (27), *p* = 0.3
Coarse crackles, *n* (%)	42 (81)	1 (33), *p* = 0.09	27 (75), *p* = 0.1	14 (93), *p* = 0.2
Fine crackles, *n* (%)	3 (5.8)	2 (67), *p* = 0.07	3 (8.3), *p* = 0.5	0 (0), *p* = 0.5
Stridor, *n* (%)	2 (3.8)	0 (0), *p* > 0.99	0 (0), *p* = 0.9	2 (13), *p* = 0.08
Diminished sounds, *n* (%)	1 (1.9)	0 (0), *p* > 0.99	0 (0), *p* = 0.3	1 (6.7), *p* = 0.3
Apnea, *n* (%)	0 (0)	0 (0)	0 (0)	0 (0)
Accessory muscle use, *n* (%)	25 (21)	2 (18), *p* > 0.99	20 (30), *p* = 0.005	4 (9.1), *p* = 0.02
SaO_2_ with room air, median (Q_1_–Q_3_) (%)^a^	96 (93–98)	96 (94–99)	94 (91–97)	98 (96–99)
Dehydration, *n* (%)	7 (5.9)	1 (9.1), *p* = 0.5	4 (6.1), *p* > 0.99	3 (6.8), *p* = 0.7
Body temperature (°C), max., median (Q_1_–Q_3_)	38.6 (38.3–39.1)	38.7 (38.2–39), *p* = 0.8	38.5 (38.3–39), *p* = 0.4	38.7 (38.3–39.3), *p* = 0.3
CRP^b^ (mg/L), max., median (Q_1_–Q_3_)^c^	18 (9–55)	13 (4–23), *p* = 0.1	15 (9–33), *p* = 0.03	42 (14–86), *p* = 0.003
Leukocyte count (E9/L), max., median (Q_1_–Q_3_)^d^	11 (7.8–14)	6.4 (4.2–12), *p* = 0.02	11 (7.9–13), *p* = 0.5	14 (7.3–19), *p* = 0.03
Chest radiograph, *n* (%)	21 (18)	1 (9.1), *p* = 0.7	11 (17), *p* = 0.8	10 (23), *p* = 0.3
Abnormal finding, *n* (%)	12 (57)	0 (0), *p* = 0.4	8 (73), *p* = 0.2	4 (40), *p* = 0.2

^a^ Information was missing from 4 cases.

^b^ CRP = C‐reactive protein.

^c^ Information was missing from 44 cases.

^d^ Information was missing from 33 cases.

The majority of infants were given symptom‐relieving medications. Paracetamol was administered to 82/119 (69%) of the infants; among these, analgesia was the indication for treatment in 42/82 (51%) cases. Of those 42 infants, 36 (86%) had good responses. As an antipyretic, paracetamol was administered to 64 infants, and of those, 44 (69%) had a good response. Ibuprofen was administered to 40/119 (34%) infants. Ibuprofen treatment was used as an analgesic in 24/40 (60%) infants, and of those, 17/24 (71%) had a good response (Table 3A).

**Table 3:** Proportions of medications and other treatment provided to the infants treated in the Pediatric Unit of Tampere University Hospital between December 27, 2019 and May 6, 2020.

	All cases (*n* = 119)	Influenza (*n* = 11)	RSV (*n* = 66)	Other etiology (*n* = 44)
**A. Treatment of pain and fever**
Paracetamol, *n* (%)	82 (69)	7 (64), *p* = 0.7	48 (73), *p* = 0.3	29 (66), *p* = 0.6
For analgesia, *n* (%)	42 (51)	5 (71), *p* = 0.4	27 (56), *p* = 0.3	11 (38), *p* = 0.08
Response, *n* (%); Yes	36 (86)	2 (40)	24 (89)	11 (100)
No	1 (2.4)	1 (20)	0	0
No information/unclear	5 (12)	2 (40)	3 (11)	0
For fever, *n* (%)	64 (78)	4 (57), *p* = 0.2	39 (81), *p* = 0.4	22 (76), *p* = 0.7
Response, *n* (%); Yes	44 (69)	3 (75)	27 (69)	15 (68)
No	4 (6.3)	0	3 (7.7)	1 (4.5)
No information/unclear	16 (25)	1 (25)	9 (23)	6 (27)
Ibuprofen, *n* (%)	40 (34)	3 (27), *p* = 0.7	23 (35), *p* = 0.8	14 (32), *p* = 0.8
For analgesia, *n* (%)	24 (60)	2 (67), *p* > 0.99	15 (65), *p* = 0.4	7 (50), *p* = 0.3
Response, *n* (%); Yes	17 (71)	0	11 (73)	6 (86)
No	2 (8.3)	0	2 (13)	0
No information/unclear	5 (21)	2 (100)	2 (13)	1 (14)
For fever, *n* (%)	33 (83)	3 (100), *p* > 0.99	18 (78), *p* = 0.7	12 (86), *p* > 0.99
Response, *n* (%); Yes	20 (61)	1 (33)	11 (61)	8 (67)
No	2 (6.1)	0	1 (5.6)	1 (8.3)
No information/unclear	11 (33)	2 (67)	6 (33)	3 (25)
Naproxen, *n* (%)	3 (2.5)	0 (0), *p* > 0.99	2 (3), *p* > 0.99	1 (2.3), *p* > 0.99
For analgesia, *n* (%)	2 (66.7)	—	2 (100), *p* = 0.3	0 (0), *p* = 0.3
Response, *n* (%); Yes	0	—	0	—
No	1 (50)	—	1 (50)	—
No information/unclear	1 (50)	—	1 (50)	—
For fever, *n* (%)	2 (67)	—	1 (50), *p* > 0.99	1 (100), *p* > 0.99
Response, *n* (%); Yes	0	—	0	0
No	0	—	0	0
No information/unclear	2 (100)	—	1 (100)	1 (100)
**B. Treatment of breathing**				
Oxygen support, *n* (%)	18 (15)	1 (9.1), *p* > 0.99	16 (24), *p* = 0.002	2 (4.5), *p* = 0.01
Response, *n* (%); Yes	16 (89)	1 (100)	15 (94)	1 (50)
No	0	0	0	0
No information/unclear	2 (11)	0	1 (6.3)	1 (50)
High‐flow nasal cannula, *n* (%)	3 (2.5)	1 (9.1), *p* = 0.3	2 (3.0), *p* > 0.99	1 (2.3), *p* > 0.99
Duration, days, median (min–max)	2 (2–4)	4	3 (2–4)	2
Response, *n* (%); Yes	3 (100)	1 (100)	2 (100)	1 (100)
No	0	0	0	0
No information/unclear	0	0	0	0
Inhaled adrenaline, *n* (%)	7 (5.9)	0 (0), *p* > 0.99	2 (3.0), *p* = 0.2	5 (11), *p* = 0.1
Response, *n* (%); Yes	6 (86)	—	1 (50)	5 (100)
No	1 (14)	—	1 (50)	0
No information/unclear	0	—	0	0
Salbutamol, *n* (%)	21 (18)	1 (9.1), *p* = 0.7	18 (27), *p* = 0.002	2 (4.5), *p* = 0.004
Response, *n* (%); Yes	14 (67)	1 (100)	11 (61)	2 (100)
No	6 (29)	0	6 (33)	0
No information/unclear	1 (4.8)	0	1 (5.6)	0
Ipratropiumbromide, *n* (%)	1 (0.8)	0 (0), *p* > 0.99	1 (1.5), *p* > 0.99	0 (0), *p* > 0.99
Response, *n* (%); Yes	0 (0)	—	0 (0)	—
No	1 (100)	—	1 (100)	—
No information/unclear	0	—	0	—
Inhaled saline, *n* (%)	9 (7.6)	1 (9.1), *p* = 0.6	9 (14), *p* = 0.004	0 (0), *p* = 0.03
Response, *n* (%); Yes	7 (78)	1 (100)	7 (78)	—
No	0	0	0	—
No information/unclear	2 (22)	0	2 (22)	—
**C. Treatment of hypovolemia and problems in eating**				
Fluid treatment, *n* (%)	25 (21)	2 (18), *p* > 0.99	15 (23), *p* = 0.6	9 (21), *p* = 0.9
Response, *n* (%); Yes	23 (92)	1 (50)	14 (93)	9 (100)
No	0	0	0	0
No information/unclear	2 (8.0)	1 (50)	1 (6.7)	0
Intravenous fluid treatment, *n* (%)	9 (7.6)	1 (9.1), *p* = 0.6	1 (1.5), *p* = 0.01	8 (18), *p* = 0.001
Response, *n* (%); Yes	9 (100)	1 (100)	1 (100)	8 (100)
No	0	0	0	0
No information/unclear	0	0	0	0
Nasogastric tube, *n* (%)	19 (16)	2 (18), *p* = 0.7	15 (23), *p* = 0.03	3 (6.8), *p* = 0.04
Response, n (%); Yes	17 (89)	1 (50)	14 (93)	3 (100)
No	0	0	0	0
No information/unclear	2 (11)	1 (50)	1 (6.7)	0
**D. Other medications**				
Antibiotics, *n* (%)	70 (59)	6 (55), *p* = 0.8	42 (64), *p* = 0.2	24 (55), *p* = 0.5
Response, *n* (%); Yes	65 (93)	6 (100)	39 (93)	22 (92)
No	0	0	0	0
No information/unclear	3 (4.3)	0	3 (7.1)	0
Unnecessary	2 (2.9)	0	0	2 (8.3)
Corticosteroids, *n* (%)	5 (4.2)	0 (0), *p* > 0.99	0 (0), *p* = 0.02	5 (11.4), *p* = 0.006
Response, *n* (%); Yes	4 (80)	—	—	4 (80)
No	1 (20)	—	—	1 (20)
No information/unclear	0	—	—	0
Oseltamivir, *n* (%)	9 (7.6)	9 (82), *p* = < 0.001	2 (3.0), *p* = 0.08	0 (0), *p* = 0.03
Response, *n* (%); Yes	8 (89)	8 (89)	2 (100)	—
No	0	0	0	—
No information/unclear	0	0	0	—
Ended due to wrong dosage	1 (11)	1 (11)	0	—

Medications to relieve breathing were also used frequently: salbutamol was given to 21/119 infants (18%), with a good response in 14/21 infants (67%) (Table 3B). Fluid resuscitation by nasogastric tube or intravenously was given to 25/119 infants (21%). Out of these, 23/25 (92%) had a good response (Table 3C). Corticosteroids were given to 5/119 infants (4.2%), all of whom were treated due to an illness with an etiology other than influenza or RSV infection. Of the infants administered corticosteroids, 4/5 (80%) had a good response (Table 3D). Out of the 11 infants who had an influenza infection, 9 (82%) received oseltamivir treatment, and 8 of them had a good response (Table 3D). Patients with influenza who did not receive oseltamivir treatment already had symptoms for over 48 h upon arrival at the hospital; therefore, oseltamivir treatment was not considered beneficial.

Overall, antibiotics were administered to 70/119 infants (59%) (Table 3D). Otitis media was the most common reason for antibiotic treatment, and amoxicillin with or without clavulanic acid was the most frequently used antibiotic (Supporting Material S2).

## 4 Discussion

The purpose of this study was to determine the treatment given to infants aged < 1 year presenting with fever and symptoms of a respiratory infection in the ED, as well as the response to the treatment. The secondary purpose was to describe the specific diagnosis behind the respiratory symptoms. The main findings were that almost all the infants received symptom‐relieving medications, and the responses to the medication were generally good. In addition, over half of the infants received antibiotics, mostly due to otitis media, with a good response. Only in a few cases was treatment with antibiotics terminated early, as it was deemed unnecessary. Moreover, bronchiolitis or bronchitis was the most common diagnosis for infants with fever and respiratory symptoms, especially among those admitted to the pediatric ward.

In our study, 42% of the infants needed hospitalization. This is in line with the findings of a prospective population‐based study that evaluated acute respiratory infections of 51,441 children aged < 18 years. In that study, 43% of children with acute respiratory infection were treated as inpatients, and infants aged < 12 months accounted for 38% of the hospitalizations [7]. In our study, the most common diagnosis among all infants was bronchiolitis or bronchitis, which were even more common among hospitalized infants. A similar finding was made in a cross‐sectional study describing epidemiological data of 66,304 children aged 1–24 months who were hospitalized due to viral respiratory infection. The study also found that the most common diagnosis was acute bronchiolitis (56.9%), and acute bronchitis was the third most common diagnosis in the study [8].

Almost all of the infants in our study received some type of symptom‐relieving medication. Paracetamol was the most used analgesic and antipyretic. Most infants showed good responses to paracetamol when used both as an analgesic and as an antipyretic, although the response was slightly better as an analgesic. In our study, pain relief was also the most common indication for paracetamol and ibuprofen treatments. This is in line with the recommendation of a 2021 National Institute for Health and Care Excellence guideline concerning fever, which stipulates that fever may be treated with antipyretics, but that the main goal of antipyretic treatment should be to relieve the child's distress [9]. The same conclusion was reached in two different reviews considering the treatment of fever in children [10, 11]. According to a 2021 systematic review, there seems to be no difference between ibuprofen and paracetamol with regard to their effectiveness in lowering body temperature; ibuprofen could possibly be better in relieving child's distress caused by fever [11]. However, ibuprofen is not recommended for infants aged < 6 months and therefore paracetamol is the main choice of antipyretic for the youngest of children [12].

In our study, antibiotics were administered to more than half of the infants. According to recommendations, uncomplicated viral respiratory tract infections should not be treated with antibiotics [13]. Therefore, it can be argued that antibiotics were used against recommendations in our study. Another Finnish register‐based study conducted between Years 2014–2020 found that doctors often prescribed antibiotics for bronchitis against guideline recommendations [14], which indicates that there is some level of misuse of antibiotic prescription present. However, the high rate of antibiotic administration in our study can partially be due to the fact that viral infections have been followed by bacterial infections. This is supported by previous findings of bacterial infections often complicating viral respiratory infections [15]. The most common indication for antibiotic treatment in our study was otitis media. Finnish guidelines recommend antibiotics for the treatment of AOM and suggest amoxicillin or amoxicillin with clavulanic acid as the first‐line antibiotics [16]. Our study followed this recommendation.

We found that treatments to relieve breathing were frequently administered. A 2014 American Academy of Pediatrics guideline for management of bronchiolitis stated that the use of bronchodilators such as salbutamol is not recommended for treatment of bronchiolitis, since they do not improve the clinical course of the illness [17]. Similar recommendation was also offered by a meta‐analysis of 13 studies that evaluated the efficiency of salbutamol in the treatment of bronchiolitis [18], as well as by a recent review published in *Lancet* concerning bronchiolitis [19]. Similarly, the 2025 Australasian Bronchiolitis Guideline advises against the use of bronchodilators in infants with bronchiolitis [20]. In our study, salbutamol was given to 18% of infants, and nearly all of those infants had RSV infection. Since RSV is the most common cause of bronchiolitis in infants [17], it can be argued that salbutamol was used against recommendation in our study. Similar findings have been reported in a previous review, which demonstrated notable variations in medication use, including bronchodilators, across and within different countries [21]. Another Finnish study, also conducted in Tays, found that between 2016 and 2020, 15% of children who were treated in the pediatric ICU due to bronchiolitis received inhaled salbutamol [22]. Therefore, using salbutamol seems to be a normal practice in our hospital despite the severity of the illness, even though the use of inhaled salbutamol in Tays has decreased significantly over the last two decades [22]. However, there is still need for improvement for the treatment to follow guidelines in this subject.

Influenza infection can be treated with antiviral drugs. Currently, out of antiviral drugs, only neuraminidase inhibitor oseltamivir is indicated in the treatment of influenza in infants < 6 months of age [23, 24]. Oseltamivir treatment should be started as early as possible after the onset of symptoms [25]. In our study, two infants with laboratory‐confirmed influenza infection did not receive oseltamivir treatment, because their symptoms had already lasted for over 48 h. The same time limit of 48 h after the onset of symptoms was implemented in a prospective Finnish study considering oseltamivir treatment of influenza in infants [26]. Our findings were in line with their results that oseltamivir treatment is beneficial in influenza‐infected children [26].

This study has a few limitations. First, the number of study participants is small. However, our study hospital has the only pediatric ED in the catchment area; therefore, our study population is a good representation of all the infants living in the area. Given that our data were collected only from tertiary‐level children's hospitals, our findings may not be generalized to all children under 1 year of age with fever and respiratory symptoms. However, this is also the population that most frequently presents to hospitals with health issues. Another limitation is that in our study, we were only able to differentiate RSV and influenza A and B infections by viral testing, which may predispose our “other etiology” group to underclassification. Testing only these three aforesaid viruses, however, was a current hospital protocol. A strength of our study is that we identified all children presenting to the ED with fever and symptoms of a respiratory infection during the study period and were able test all these infants for these three viruses. Due to the retrospective nature of our data, some medication responses were undocumented, potentially biasing the results. However, given the limitations of retrospective data, the primary data collection and participant identification in our study were conducted prospectively.

## 5 Conclusions

Our findings indicate that nearly half of the infants needed hospitalization, and symptom‐relieving medications, such as analgesics, were a key form of treatment, with generally good responses. Although treatment guidelines advice against antibiotic treatment for viral infections and majority of the infants in our study had a laboratory confirmed viral infection, antibiotics were used frequently. However, otitis media was the most common indication for antibiotic treatment, supporting the previous understanding that viral respiratory infections are often complicated by secondary bacterial infections.

## Author Contributions

**Sallamaria Länsisalo:** writing – original draft, writing – review and editing. **Paula Heikkilä:** writing – review and editing, methodology, conceptualization. **Sauli Palmu:** writing – review and editing, methodology, conceptualization. All authors have read and approved the final version of the manuscript.

## Ethics Statement

The data were gathered from the hospital's patient records and families were not contacted for this study. No identifiable information is reported.

## Consent

The written informed consent was waived by the Ethics committee, who waived the informed consent due to the Finnish legislation that does not require informed consents when the patients are not contacted.

## Conflicts of Interest

The authors declare no conflicts of interest.

## 1 Transparency Statement

The manuscript guarantor (Sauli Palmu) affirms that this manuscript is an honest, accurate, and transparent account of the study being reported; that no important aspects of the study have been omitted; and that any discrepancies from the study as planned (and, if relevant, registered) have been explained.

## Supporting information

Supporting material R1.

## Data Availability

The anonymous data sets used and/or analyzed during the current study are available from the corresponding author on reasonable request. The corresponding author had full access to all of the data in this study and takes complete responsibility for the integrity of the data and the accuracy of the data analysis.
