# Supplementary material for: Management of Infants Treated for Respiratory Viral Infections: A Finnish Retrospective Register‐Based Study
Source: Health Sci Rep. 2025 Oct 28;8(11):e71414. doi: 10.1002/hsr2.71414 (PMC12560114; doi:10.1002/hsr2.71414)
Supplement: Supplementary file 1 — Supporting material R1. [file HSR2-8-e71414-s001.docx]

Article title: Management of Infants Treated for Respiratory Viral Infections: A Finnish Retrospective Register-based Study

Author names: Sallamaria Länsisalo, M.D^1^, Paula Heikkilä, PhD^2^, Sauli Palmu, MD, PhD^2^

^1^Tampere University, Faculty of Medicine and Health Technology, ^2^Tampere Centre for Child, Adolescent and Maternal Health Research, Faculty of Medicine and Health Technology, Tampere University and University Hospital, Tampere, Finland

Address for correspondence: Sallamaria Länsisalo, Tampere Centre for Child Health Research, Arvo Ylpön katu 34, FI-33014, University of Tampere, Tampere, Finland

sallamaria.kelloniemi@tuni.fi

***Supplementary Material 1.* Proportions of final diagnoses of infants treated for symptoms of respiratory infection at Pediatric Unit of Tampere University Hospital.**

|  | All cases (n=119) | Patients treated in outpatient clinic (n=69) | Patients treated in hospital (n=50) |
| --- | --- | --- | --- |
| Acute upper respiratory tract infection, n (%) | 32 (27) | 23 (33) | 9 (18) |
| Bronchiolitis and bronchitis, n (%) | 51 (43) | 23 (33) | 28 (56) |
| Influenza, n (%) | 10 (8.4) | 6 (8.7) | 4 (8.0) |
| COVID-19 or suspected case, n (%) | 3 (2.5) | 0 (0) | 3 (6.0) |
| Pneumonia, n (%) | 5 (4.2) | 0 (0) | 5 (10) |
| Otitis media, n (%) | 45 (38) | 22 (32) | 23 (46) |
| Shortness of breath, n (%) | 1 (0.8) | 1 (1.4) | 0 |
| Fever and febrile convulsions, n (%) | 5 (4.2) | 2 (2.9) | 3 (6.0) |
| Sepsis or other unspecified bacterial infection, n (%) | 2 (1.7) | 0 (0) | 2 (4.0) |
| Other_†_, n (%) | 11 (9.2) | 2 (2.9) | 9 (18) |
| _†_*Diagnoses such as conjuctivis, pyelonephritis and lymphadenitis.* | | | |

***Supplementary Material 2.* Proportions of different antibiotics and the indications to antibiotic treatment administered to infants treated for symptoms of respiratory infection in the Pediatric Unit of Tampere University Hospital.**

|  | All cases (n=119) | Influenza (n=11) | RSV (n=66) | Other aetiology (n=44) |
| --- | --- | --- | --- | --- |
| Antibiotic, n (%) | 70 (59) | 6 (55), p=0.8 | 42 (64), p=0.23 | 24 (55), p=0.5 |
| Amoxicillin ± clavulanic acid, n (%) | 55 (79) | 3 (50) | 39 (93) | 14 (58) |
| Parenteral antibiotic treatment, n (%) | 11 (16) | 2 (33) | 2 (4.8) | 7 (29) |
| Cephalosporin, n (%) | 17 (24) | 2 (33) | 5 (12) | 11 (46) |
| Other antibiotics, n (%) | 4 (5.7) | 2 (33) | 1 (2.4) | 2 (8.3) |
| Indication for antibiotic treatment |  |  |  |  |
| Otitis media, n (%) | 51 (73) | 3 (50) | 39 (93) | 10 (42) |
| Lower respiratory tract infection, n (%) | 8 (11) | 1 (17) | 6 (14) | 2 (8.3) |
| Upper respiratory tract infection, n (%) | 1 (1.4) | 0 (0) | 0 (0) | 1 (4.2) |
| Urinary tract infection, n (%) | 8 (11) | 1 (17) | 0 (0) | 7 (29) |
| Generalized bacterial infection, n (%) | 7 (10) | 1 (17) | 2 (4.8) | 4 (17) |
| Conjuctivitis, n (%) | 3 (4.3) | 1 (17) | 0 (0) | 0 (0) |
| Indication unclear, n (%) | 1 (1.4) | 1 (17) | 0 (0) | 0 (0) |
